# Identification of Quantitative Trait Loci (QTLs) and candidate genes for trichome development in *Brassica villosa* using genetic, genomic, and transcriptomic approaches

**DOI:** 10.1007/s00438-024-02223-5

**Published:** 2025-01-07

**Authors:** Thomas Bergmann, Wanzhi Ye, Steffen Rietz, Daguang Cai

**Affiliations:** 1 Department of Molecular Phytopathology and Biotechnology, Institute of Phytopathology, Christian-Albrechts-University of Kiel, 24118 Kiel, Germany; 2 NPZ Innovation GmbH, 24363 Holtsee, Germany; 3 Present Address: School of Biological Sciences, The University of Western Australia, Perth, Western Australia 6009 Australia

**Keywords:** Trichomes, *Brassica villosa*, Genetic mapping, QTL analysis, *TRY*, Genomics and transcriptomics of *Brassica*

## Abstract

*Brassica villosa* is characterized by its dense hairiness and high resistance against the fungal pathogen *Sclerotinia sclerotiorum*. Information on the genetic and molecular mechanisms governing trichome development in *B. villosa* is rare. Here, we analyzed an F_2_ population, derived from a cross between *B. villosa* and the glabrous *B. oleracea* by QTL mapping and transcriptomic analyses. As a result, the phenotyping of 171 F_2_ progenies revealed a wide range of variation in trichome development. Subsequent genotyping with the 15-k Illumina SNP array resulted in a genetic map with 970 markers and a total length of 812 cM. Four QTLs were identified, which explained phenotypic variation from 3.2% to 40.3%. Interestingly, one of these was partially co-localized with the major QTL for Sclerotinia-resistance previously detected in the same F_2_ population. However, only a moderate correlation between trichomes and Sclerotinia-resistance was observed. In total, 133 differentially expressed genes (DEGs) associated with trichome development were identified, from which only *BoTRY*, an orthologue of Arabidopsis *TRY* encoding a MYB transcription factor negatively regulating trichome development, was located within the major QTL. Expression of *BoTRY* was tissue-specific and highly variable between the hairy and glabrous species, suggesting that *BoTRY* may also act as a master-switch regulator of trichome development in *B. villosa*. This study provides valuable data for further understanding the genetic architecture of trichome development and identifying related genes and mechanisms in *Brassica* species*.* Molecular markers can be developed to facilitate the introgression and selection of this trait in oilseed rape breeding.

**Supplementary Information:**

The online version contains supplementary material available at 10.1007/s00438-024-02223-5.

## Introduction

Trichomes are hairy-like, outgrowing structures from the epidermis of plant cells. They appear in various forms with different features (Johnson 1975). Trichomes form a protective or defensive layer on the plant surface against a multitude of biotic and abiotic threats, such as herbivores, pathogens, UV damage, excessive transpiration, and metal intoxication (Wang et al. 2021; Li et al. 2023). Depending on their metabolical activity, they can be categorized into glandular and non-glandular trichome types. Glandular trichomes are usually multicellular and can biosynthesize, secrete, and store secondary metabolites, whereas non-glandular trichomes are commonly unicellular with no biochemically active capabilities (Li et al. 2023). Antimicrobial phenolics accumulate in the cell walls of the non-glandular trichomes building a physical and/or chemical barrier defending against biotic stress (Karabourniotis et al. 2020). The underlying genetic and molecular machanisms are largely unknown. Identifying new trichome sources and genes involved in trichome development in *Brassica* species is of great interest to breeders seeking to improve plants’ defences against pathogen attacks.

*Brassica villosa* is a densely-haired species in the *Brassica* complex (C cytodem). Layers of hair-like non-glandular trichomes protect plants from various pests, such as the pollen beetle, cabbage aphids, and the cabbage white fly (Nayidu et al. 2014b). *B*. *villosa* exhibits high resistance against the fungus *Sclerotinia sclerotiorum* (Mei et al. 2011; Taylor et al. 2018). The QTLs for Sclerotinia-resistance have been recently identified in *B. villosa* (Bergmann et al. 2023), while so far no information is available on the genetic basis and molecular mechanisms for trichome development. In *A. thaliana*, the formation of branched, non-glandular trichomes is controlled by a complex network of transcription factors, including the positive enhancers *GL1*, *GL2*, *GL3*, *EGL3*, and *TTG1*, as well as the negative regulator *TRY* (Balkunde et al. 2010). Consistently, high expression profiles of *GL1*, *GL2*, *EGL3*, and *TTG1* were identified in the densely-haired *B. villosa*. subsp. *drepanensis* by targeted gene analyses (Nayidu et al. 2014b, a). However, several orthologous genes of the negative regulatory genes, such as *TRY* and *CPC*, were also highly expressed in the *B. villosa* accession compared to glabrous *Brassica* species (Nayidu et al. 2014b, a). In the hairy *B. incana*, *TRY* was identified as a major candidate for leaf trichome formation and exhibited a lower expression level than in the glabrous *B. oleracea* cultivar (Mei et al. 2017). This discrepancy in the expression of trichome-related genes may contribute to their different modes of trichome development, highlighting their diverse and complex mechanisms in the *Brassica* family (Nayidu et al. 2014b; Mei et al. 2017). It is believed that *B. villosa* subsp. *drepanensis* accumulates high levels of metals in its trichomes which correlates with the metal enrichment-related genes contributing to tolerance to salinity (Scialabba et al. 1999; Nayidu et al. 2014b). The accumulation of metals and two alkaloid-like compounds in the trichomes of this *Brassica* species may provide an additional chemical defense layer against insect pests (Nayidu et al. 2014b). Although non-glandular trichomes have been reported to form an effective defense layer against insects in *Brassica* species (Obermeier et al. 2022), their role in defence against fungal pathogens such as *S. sclerotiorum* has not yet been described.

In this study, we investigated an F_2_ population derived from a cross between the hairy *B. villosa* (BRA1896) and a glabrous *B. oleracea* accession (BRA1909) aiming at the exploration of the genetic basis and molecular mechanisms controlling trichome development in *B. villosa* by using genetic, genomic, and transcriptomic approaches. We report four QTLs and 133 differentially expressed genes (DEGs) associated with trichome development in *B. villosa.* We found that *BoTRY,* an orthologue of Arabidopsis *TRY* for a MYB transcription factor that negatively regulates trichome development in Arabidopsis, is located within the major QTL, thus leading to the assumption that *BoTRY* may also be a crucial factor regulating trichome formation in *B. villosa.* In addition, a partial co-localization of the major QTLs for trichomes and Sclerotinia-resistance was identified, and a moderate correlation between the two traits was observed. To our knowledge, this is the first report on genetic, genomic, and transcriptomic analyses on trichome development in the hairy *B. villosa* of the C cytodeme, and provides valuable data for further understanding the genetic architecture of trichome development and identifying related candidate genes and mechanisms as well as functions in *Brassica* species. Molecular markers can be developed to facilitate the introgression and selection of this important trait in oilseed rape breeding, making *B. villosa* even more unique and valuable.

## Materials and methods

### Plant material and screening of trichome phenotypes in the F_2_ population

The wild Brassica species *Brassica villosa* (BRA1896) and *Brassica oleracea* (BRA1909) used for this study belong to the C cytodeme of the *Brassica* complex with a chromosome number of *2n* = 18 (Warwick et al. 2009). A segregating F_2_ population (Population B) derived from an interspecific cross between these two species was previously generated (Bergmann et al. 2023), which showed a wide range of variation in the trichome phenotypes. Population B consisted of 184 F_2_ individuals, of which 171 F_2_ individuals were available at the time of the trichome screening. All the 171 F_2_ individuals and *Brassica* species (BRA1896, BRA1909) were screened for Sclerotinia-resistance previously by the detached leaf- and petiole-assay, respectively (Bergmann et al. 2023). The leaf- and petiole assays were performed in triplicate. This enabled us to link the trichome phenotype of each F_2_ individual to its leaf- and petiole-lesions. Trichome phenotyping was based on the method described by Mei et al. (2017) with a modification. In this study, trichome phenotypes were classified into five groups ranging from “0” (completely hairless) to “4” (mostly densely-haired). F_2_ individuals were scored according to the intensity of the trichome layer on the leaf and petiole, respectively. Each tissue (leaf or petiole) with visible trichomes was given a score of one or two if a denser trichome layer was observed. Thus, the maximal score for each F_2_ individual was 4, which was classified as the group “4”.

### Mapping genetic loci for trichome phenotypes

The genotyping and the genetic map construction were described by Bergmann et al. (2023) in detail. Briefly, DNA isolated from leaves of both *Brassica* species and F_2_ individuals using the cetyltrimethylammonium bromide (CTAB) method (Rogers and Bendich 1985) was adjusted to 20 ng/µl. The adjusted DNA was sent in two 96-wells to TraitGenetics GmbH (Gatersleben, Germany) for genotyping with the Brassica 15-k Illumina SNP chip with a total of 13,714 SNPs. For this study, we used the genotypic data of the 171 F_2_ individuals from Population B to construct a genetic map. The SNP sequences were assigned to their most likely physical positions in the *B. oleracea* (*cv*. TO1000) reference genome (Parkin et al. 2014) via the BLAST + software (Altschul et al. 1990; Camacho et al. 2009). The genetic map was constructed with the R/qtl package (Broman et al. 2003) and illustrated with the LinkageMapView package (Ouellette et al. 2018). The QTL analysis was performed with the R/qtl package (Broman et al. 2003) according to the workflow described in Broman and Sen (2009). First, we conducted a single-QTL scan with a non-parametric phenotype model considering the rank-based phenotypes since the trichome phenotypes were not normally distributed. This step was repeated with the EM algorithm, the Haley-Knott regression, and the extended Haley-Knott method, assuming a normal phenotype model. The results from the different methods were compared. Second, we used the Haley-Knott regression as a method for further analyses as it is more robust and supports the analysis of extended QTL models (Broman and Sen 2009). The peak marker of the most significant QTL was used as a covariate in the single-QTL model to scan for additive and interactive effects of this marker to other loci. Third, we conducted a two-dimensional QTL scan. Loci that showed significant *P* values in the covariate single-QTL scan and in the two-dimensional QTL scan were considered as additional QTLs. Finally, a multiple-QTL model was set up according to the identified loci from the previous scans and used to refine their most likely QTL positions with maximum likelihood by an iterative scan within the context of a fixed multiple-QTL model. The multiple-QTL model was used to estimate the QTL effects and the amount of phenotypic variance they explained based on variance analysis (ANOVA). The significance thresholds were determined via genome-wide scan adjusted *P* values based on permutation tests (10,000 permutations for single-QTL scans; 2000 permutations for two-dimensional QTL scans).

### Association analysis of trichomes and Sclerotinia-resistance

A detailed description of the leaf- and petiole-inoculation method is available in Bergmann et al. (2023). Briefly, for each F_2_ plant, two leaves and two petioles were infected in three independent experiments, according to Mei et al. (2011) and Zhao et al. (2004) and the lesion sizes were averaged. The fungus was cultured on potato dextrose agar (PDA; 20 g/l PDB, 15 g/l Bacto agar) plates with a pH of 5.6 at 21 °C and transferred to a new PDA plate two days before inoculation. The lesion values were used to assess whether the types of hairiness, represented by the trichome groups, are associated with different levels of Sclerotinia-resistance. The statistical analyses were performed with the R software (R Core Team 2023). The analyses were based on linear models with residuals assumed to be normally distributed and homoscedastic based on graphical residual analysis. First, the association between the trichome groups and the level of Sclerotinia-resistance was evaluated for each infection experiment and assay type with the Spearman’s correlation coefficient, followed by the stepwise test of Williams to assess whether an overall monotonic trend relationship between the trichome groups and the lesion sizes could be observed (That is, if an increase in hairiness is associated with a decrease in lesion sizes). Second, a type III one-way ANOVA was performed between the lesion sizes of the trichome groups, followed by post-hoc multiple comparisons between the lesion sizes of the trichome groups to the grand mean of all trichome groups with the multcomp package (Hothorn et al. 2008). Figures were created with the ggplot2 package (Wilkinson 2011).

### Screening of candidate genes for trichome formation

The physical positions of the SNP markers in the *B. oleracea* TO1000 reference genome were used to identify the QTL regions and candidate genes involved in trichome formations with support of the annotation of the reference genome and RNAseq data previously generated by Bergmann et al. (2023) using the same species *B. villosa* (BRA1896) and *B. oleracea* (BRA1909). The RNA used was isolated from Sclerotinia-inoculated and mock-treated petioles from three independent biological replications. Although the RNAseq experiments had been designed for identifying candidate genes for Sclerotinia-resistance, DEGs related to trichome development in *B. villosa* can be accessed from the datasets, e.g. from the mock-treated samples. Here, the gene expression was measured by the fragments per kilobase per million (FPKM) value and compared only between the mock-treated control samples of *B. villosa* and *B. oleracea*. Library preparation and RNA sequencing were performed at Novogene (Hong Kong, China) with the Illumina HiSeq 4000 system. Raw reads were filtered by quality and aligned to the *B. oleracea* TO1000 reference genome and assembled to a transcriptome according to Pertea et al. (2016) with HISAT2 (Kim et al. 2019) and StringTie (Pertea et al. 2015). Reads that could neither be aligned to the *B. oleracea* reference genome nor to the *S. sclerotiorum* 1980 reference genome (Amselem et al. 2011) were assembled de novo to transcripts by Trinity (Grabherr et al. 2011). Genes were annotated with the TransDecoder software (https://github.com/TransDecoder/TransDecoder/wiki) and by a BLAST + search against the *B. napus* and *A. thaliana* RefSeq databases. DEG analysis was performed via DESeq2 (Love et al. 2014) between the mock-treated samples of BRA1896 and BRA1909. Genes were considered to be statistically differentially expressed with an adjusted *P* value ≤ 0.05 and annotated to Gene Ontology (GO) terms via the KOBAS database from Xie et al. (2011). The detailed annotation workflow for the RNAseq data is available in Bergmann et al. (2023). The GO enrichment analysis was performed with the goseq package (Young et al. 2010) and *P* values were adjusted via the FDR method by Benjamini and Hochberg (1995). DEGs linked to trichome-related processes were analyzed and visualized with the ComplexHeatmap package by Gu et al. (2016).

### Expression analysis of candidate genes

Total RNA was isolated from leaves of *B. villosa* and *B. oleracea* with three biological replications, respectively. cDNA was synthesized from 1 µg total RNA treated with RNAse-free DNase I (Thermo Fisher Scientific, Massachusetts, USA) in a volume of 20 µl with the RevertAid First Strand cDNA Synthesis Kit (Thermo Fisher Scientific, Massachusetts, USA) according to the manufacturer’s instructions. Primers were designed based on the reference gene annotation of the *B. oleracea* TO1000 genome (Table S5). Two microliters cDNA (1:5 diluted) were mixed with 18 µl MasterMix according to the manual of the qPCRBIO SyGreen Mix (PCR Biosystems Inc., Pennsylvania, USA). RT-qPCR was performed on a CFX96 Touch Real-Time PCR Detection System (Bio-Rad Laboratories, California, USA) under the following conditions: 3 min 95 °C; 45 × 10 s 95 °C, 10 s 59 °C, 10 s 72 °C; 10 s 95 °C, melting curve from 65 °C to 95 °C. Gene expression level was determined using the delta CT Method and calculated in relation to the reference gene *BoACT7* (Bo3g005290) that is stably expressed in the two species (Bergmann et al. 2023). Each measurement consisted of three independent biological replicates incorporating three technical replicates each. The relative expression was log_2_-transformed. An ANOVA was conducted and followed by multiple contrast tests with the multcomp package (Hothorn et al. 2008).

## Results

### Trichome variation in the F_2_ mapping population

**Fig. 1:**
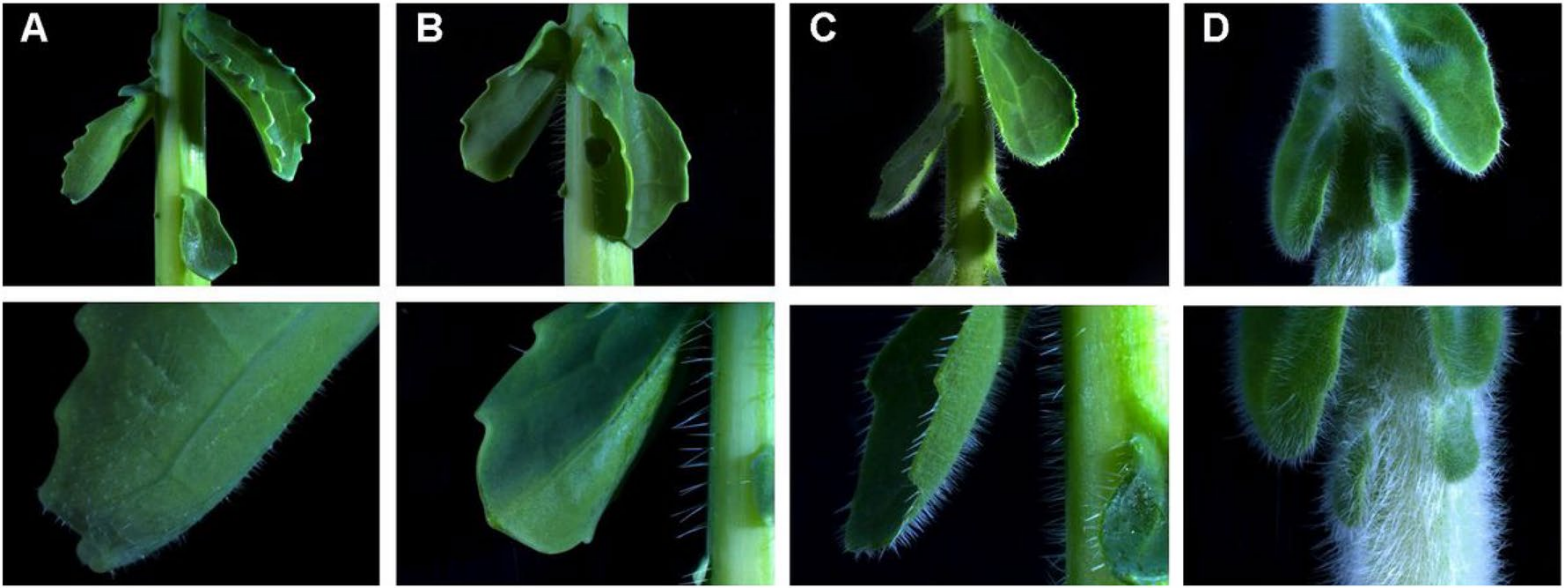
Exemplary representation of the trichome variation in the F_2_ population from a cross between the hairy *B. villosa* (BRA1896) and the glabrous *B. oleracea* (BRA1909). The pictures show the crossover from petiole to leaf. The hairiness of petioles is representative for the hairiness of stems. **A**–**C** Representative trichome phenotypes of F_2_ plants. **D** Trichome phenotype of the hairy *B. villosa*

**Fig. 2:**
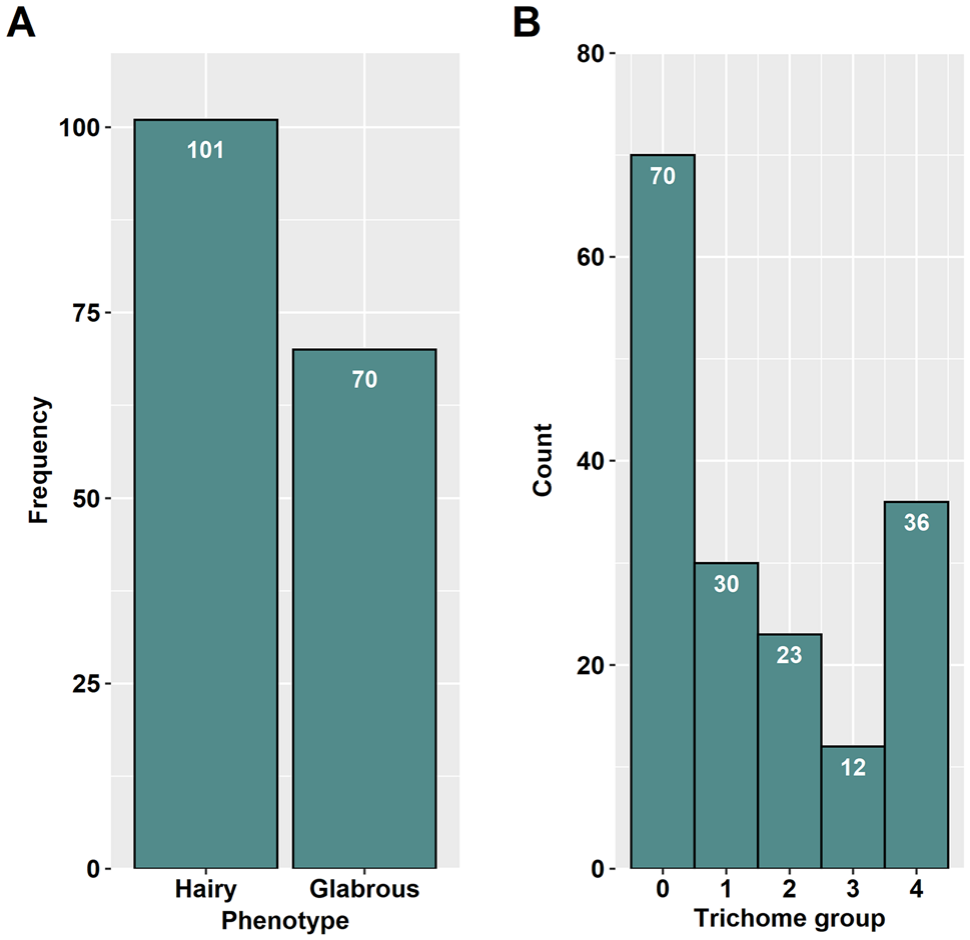
**A** Distribution of hairy and glabrous F_2_ plants. The distribution shows a significant deviation from a monogenic 3:1 segregation (X^2^ = 23.16, P value < 0.001). **B** Classification of trichome phenotypes into five trichome groups ranging from group “0” (glabrous) to group “4” (most densely haired) based on trichome density and covered tissue

The F_2_ population showed a broad range of trichome variation in density and haired tissue as well. As shown in Fig. 1, some F_2_ individual plants exhibited a sparse layer with short trichomes on the leaves only (Fig. 1A), while others had a dense layer with long trichomes on the petioles (Fig. 1B). Some F_2_ plants showed a dense layer with long trichomes on both leaves and petioles (Fig. 1C). However, no F_2_ individual plant was as hairy as *B. villosa* (Fig. 1D). In total, 101 F_2_ plants were classified to hairy and 70 to glabrous (Fig. 2A). The broad range of variation indicated that the trichome phenotype is a quantitative trait in *B. villosa.* This is supported by its significant deviation from a monogenic 3:1 segregation (*X*^*2*^ = 23.16, *P* value < 0.001). Therefore, we classified the F_2_ plants into five categories according to their trichome density and the amount of covered tissue from the groups “0” (glabrous) to “4” (most densely haired). Of the 101 hairy F_2_ individuals, 30 were assigned to group "1", 23 to the group “2”, 12 to the group “3”, and 36 to the group “4” (Fig. 2B).

### Construction of the genetic map

The 15-k Illumina SNP-array carrying 13,714 single nucleotide polymorphism (SNP) markers was used for genetic map construction. Of these SNP markers, 3124 (22.78%) were polymorphic while 6443 (46.98%) were monomorphic in the F_2_ population and 4147 SNPs (30.24%) failed to be detected (Supplementary Fig. 1). A set of 988 SNPs showed an expected 1:2:1 segregation and after quality filtering it was reduced to 970 unique polymorphic (bin) SNPs that resulted in a genetic map (Supplementary File 1). The SNPs were arranged into 10 linkage groups with a total length of 812 cM and an average spacing of 0.8 cM (Supplementary Table 1). The 10 linkage groups corresponded to the nine chromosomes of the *B. oleracea* TO1000 reference genome with two linkage groups (C04a, C04b) representing the beginning and ending of chromosome C04 due to a low SNP coverage in the middle of the chromosome. The genetic positions of the SNP markers were in accordance with their physical position in the *B. oleracea* TO1000 reference genome (Supplementary Data 1).

### Mapping genomic loci for hairiness in *B. villosa*

Three QTLs for the trichome phenotype were identified in the F_2_ population with the single-QTL scan (Table 1). They were identified on linkage groups C01 (tQTL1), C04b (tQTL2), and C09 (tQTL3) with all tested phenotype models. Differences between the QTL methods were given in tQTL2 on linkage group C04b regarding the QTL peak position. The non-parametric QTL scan detected the peak position at 3 cM with a LOD of 3.7 (*P* value = 0.03), while the other three methods detected the peak position at 20.1 cM with a LOD of 3.8 (*P* values < 0.05). The two-dimensional QTL scan (Haley-Knott regression) supported the peak position at 3 cM (LOD_av1_ = 6.68, *P* value < 0.001) and was further strengthened by a fixed multiple-QTL model analysis. The peak of tQTL1 (LOD = 14.75, *P* value < 0.001) at position 51 cM between the markers “Bn-scaff_15747_1-p105633” (14,270,425 bp) and “Bn-scaff_22790_1-p152675” (16,593,775 bp) explained 40.3% of phenotypic variance. Genotypes with *B. villosa* alleles showed an average trichome phenotype of 3.53 (standard error: ± 0.2), while genotypes with *B. oleracea* alleles only showed an average trichome phenotype of 0.36 (standard error: ± 0.2) at this locus. The tQTL2 on C04b explained 7.5% of phenotypical variance with its peak (LOD = 3.7, *P* value = 0.03) at “Bn-A04-p12917949” (44,795,007 bp) between the flanking markers “Bn-A04-p12333381” (44,315,666 bp) and “Bn-A04-p13241959” (45,357,144 bp). The average phenotype at this locus for genotypes with *B. villosa* alleles was 2.46 (standard error: ± 0.42) while for genotypes with *B. oleracea* alleles was 1.02 (standard error: ± 0.18). The tQTL3 on linkage group C09 explained 13% of phenotypical variance with the QTL peak (LOD = 8.17, *P* value < 0.001) at 6 cM between the flanking markers “Bn-scaff_17526_1-p1667260” (2,112,449 bp) and “Bn-scaff_17526_1-p952618” (2,827,091 bp). Genotypes with *B. villosa* alleles showed an average phenotype of 2.38 (standard error: ± 0.22), while genotypes with *B. oleracea* alleles showed an average phenotype of 0.38 (standard error: ± 0.22).

**Table 1:** Identified QTLs for trichomes in the F_2_ population

QTL	Scan*	LOD	Linkage group	Position [cM]	Marker^a^	SNP (hairy/glabrous)	*P* value	Var [%]	Add^b^	Dom^c^	
tQTL1	1D	14.75	C01	51	Bn-scaff_15747_1-p105633	A/G	< 0.001	40.3	1.54	– 0.67	
tQTL2	1D	3.7	C04b	3	Bn-A04-p12917949	C/T	0.03	7.5	0.77	– 0.13	
tQTL3	1D	8.2	C09	6	Bn-scaff_17526_1-p1667260	C/T	< 0.001	13	0.83	0.13	
tQTL4	2D	4	C03	54.4	Bn-scaff_16352_1-p1178947	C/T	0.004	3.2	0.4	0.29	

^*^1D = single-QTL scan with a non-parametric model; 2D = two-dimensional QTL scan with the Haley-Knott regression

^a^Marker at peak or nearest to the QTL peak

^b^Additive effect. Positive values indicate alleles from *B. villosa* (BRA1896) increase the hairiness

^c^Dominance effect. Positive values indicate alleles from *B. villosa* are dominant

*LOD* Logarithm of the odds, *cM* centiMorgan, *Var* Proportion of explainable variance

**Fig. 3:**
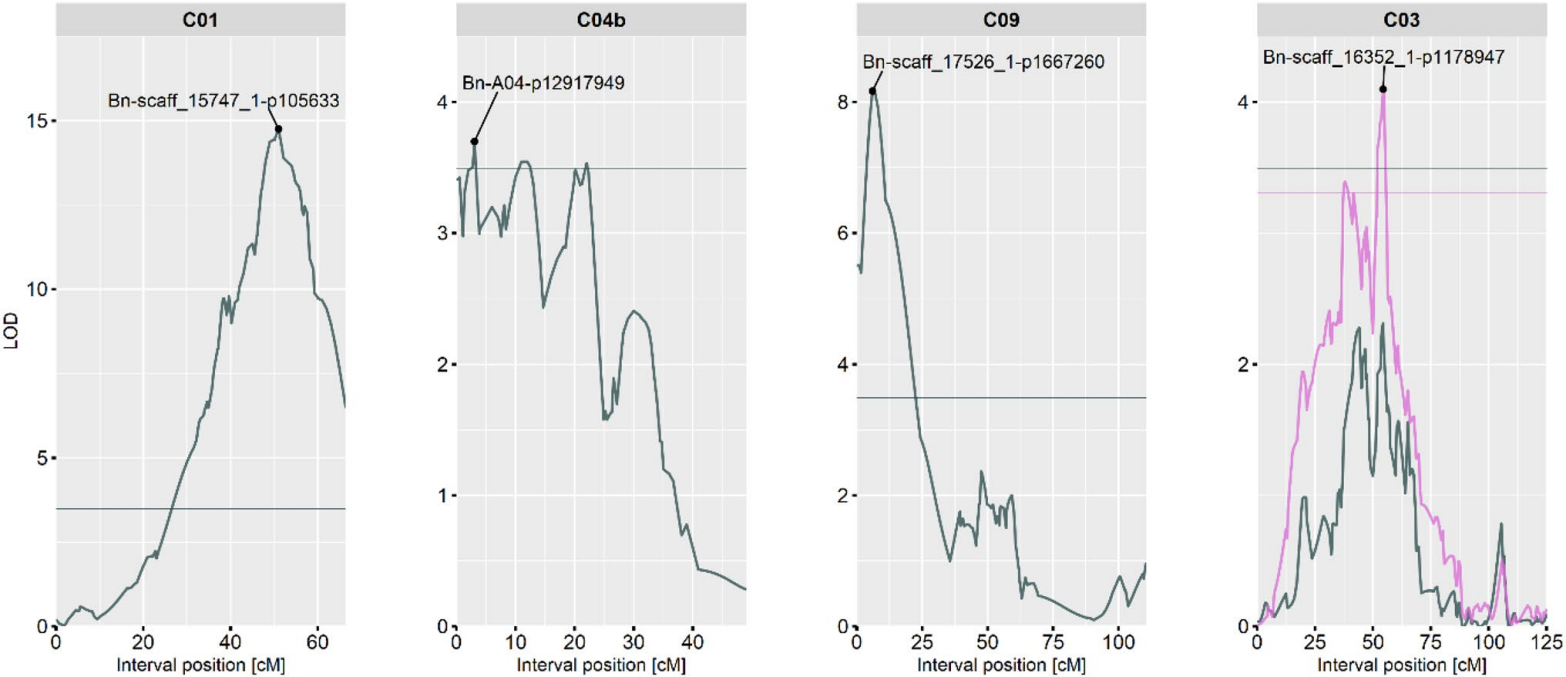
LOD profiles for genomic loci associated with the trichome phenotype in the F_2_ population. Horizontal lines indicate significance thresholds. The QTL peaks are highlighted by the nearest SNP marker. Dark gray: LOD profile from the non-parametric single-QTL scan. Purple: LOD_av1_ profile for chromosome C03 from the two-dimensional parametric QTL scan (Haley-Knott regression) with the tQTL1 on chromosome C01 at position 51 cm (Bn-scaff_15747_1-p105633) as covariate

In addition, both the single-QTL scan with tQTL1 as covariate and the two-dimensional QTL scan indicated one locus on the linkage group C03 (tQTL4) with additive effects (LOD_av1_ = 4.01, *P* value = 0.004). The peak was detected at position 54.4 cM between the markers “Bn-scaff_16352_1-p1063388” (17,865,682 bp) and “Bn-scaff_16352_1-p1178947” (17,981,241 bp) explaining 3.2% of phenotypical variance. The average phenotype was 1.78 (standard error: ± 0.20) for genotypes with *B. villosa* alleles and 0.67 (standard error: ± 0.28) for genotypes with *B. oleracea* alleles. The alleles of the hairy *B. villosa* were dominant in tQTL3 and tQTL4. Figure 3 illustrates the LOD profiles for the QTLs. LOD values of the single-QTL scans for all linkage groups and their corresponding QTL interval estimates are provided in Supplementary Data 2.

### Co-localization of the major QTLs for trichomes and for Sclerotinia-resistance on chromosome C01

**Fig. 4:**
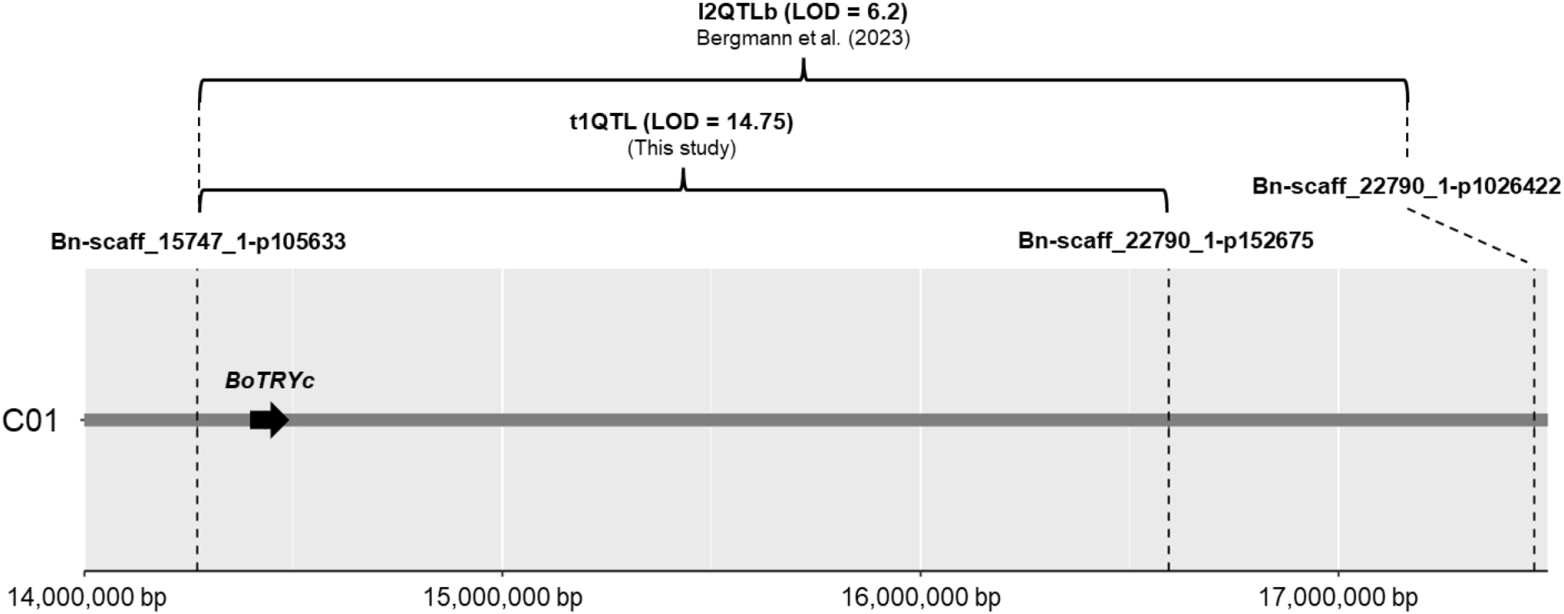
Positional overview of the co-localization of the major QTL for trichomes (t1QTL) with the major QTL for Sclerotinia-resistance (l2QTLb) from Bergmann et al. (2023). The brackets highlight the peak intervals of the QTLs. The arrow indicates the position of *BoTRYc*

We identified that the flanking marker “Bn-scaff_22790_1-p152675” of tQTL1 at 16,593,775 bp on chromosome C01 in the *B. oleracea* TO1000 genome is also the peak marker of the major QTL for Sclerotinia-resistance (l2QTLb) previously detected in this F_2_ population (Bergmann et al. 2023) (Fig. 4). The peak of l2QTLb was detected at the marker “Bn-scaff_22790_1-p152675” (16,593,775 bp) between the left flanking marker “Bn-scaff_15747_1-p105633” (14,270,425 bp) and the right flanking marker “Bn-scaff_22790_1-p1026422 (17,467,522 bp). These data indicate a partial overlapping of the major QTLs for trichomes and for Sclerotinia-resistance in *B. villosa*.

To confirm this, we included the phenotyping data of Sclerotinia-infection, generated previously with the same F_2_ population (Bergmann et al. 2023), in the analysis. The leaf- and petiole-lesion data were available for all 171 F_2_ individuals used for trichome phenotyping in this study. The Spearman’s rank correlation analysis revealed significantly negative correlations between the lesion values and the hairiness in the 1st (*P* value < 0.02) and 2nd (*P* value < 0.001) leaf-assay experiments, as well as negative correlations in the 1st (*P* value 0.09) and 2nd (*P* value 0.08) petiole-assay experiments although these were less significant (Table 2). The amount of explainable variance, measured by the coefficient of determination (*R*^2^), was low across all assays and ranged from −9% to 1%. The Williams’ test aligned with the results of Spearman’s correlation analysis and showed that hairy trichome groups had, on average, lower lesion values in comparison to the glabrous trichome group across all experiments and assays except for the 3rd petiole-assay, where hairy trichome groups tended to higher lesion values (Supplementary Table 2). Accordingly, the most significant trend relationship between the lesion values and the trichome groups was given in the 2nd leaf assay, where the most densely haired genotypes of the group “4” showed on average 199 mm^2^ smaller leaf lesion values than glabrous genotypes of the group “0” (*P* value < 0.001) and the stepwise mean of all hairy trichome groups was significantly less than that of the group “0” (*P* values < 0.001). Further, group “4” showed significantly lower lesion values compared to the grand mean of all groups in three of the six assays (Supplementary Fig. 2, Supplementary Table 3). Tukey’s all-pair comparison revealed that trichome group “4” showed the most significant differences from the other trichome groups (Supplementary Fig. 2). These data suggest a trend association existing between Sclerotinia-resistance and trichomes in *B. villosa*.

**Table 2:** Spearman’s rank correlation analysis between the trichome phenotypes and lesion values

Experiment	Assay	*R*^*2*^	*rho*	*P* value
1st	Leaf	– 0.033	– 0.182	**0.0172**
2nd	Leaf	– 0.095	– 0.309	** < 0.001**
3rd	Leaf	– 0.001	– 0.028	0.721
1st	Petiole	– 0.016	– 0.128	**0.095**
2nd	Petiole	– 0.020	– 0.141	**0.076**
3rd	Petiole	0.010	0.098	0.206

*R*^*2*^ Coefficient of determination

### Identification of candidate genes for trichome formation in *B. villosa*

**Fig. 5:**
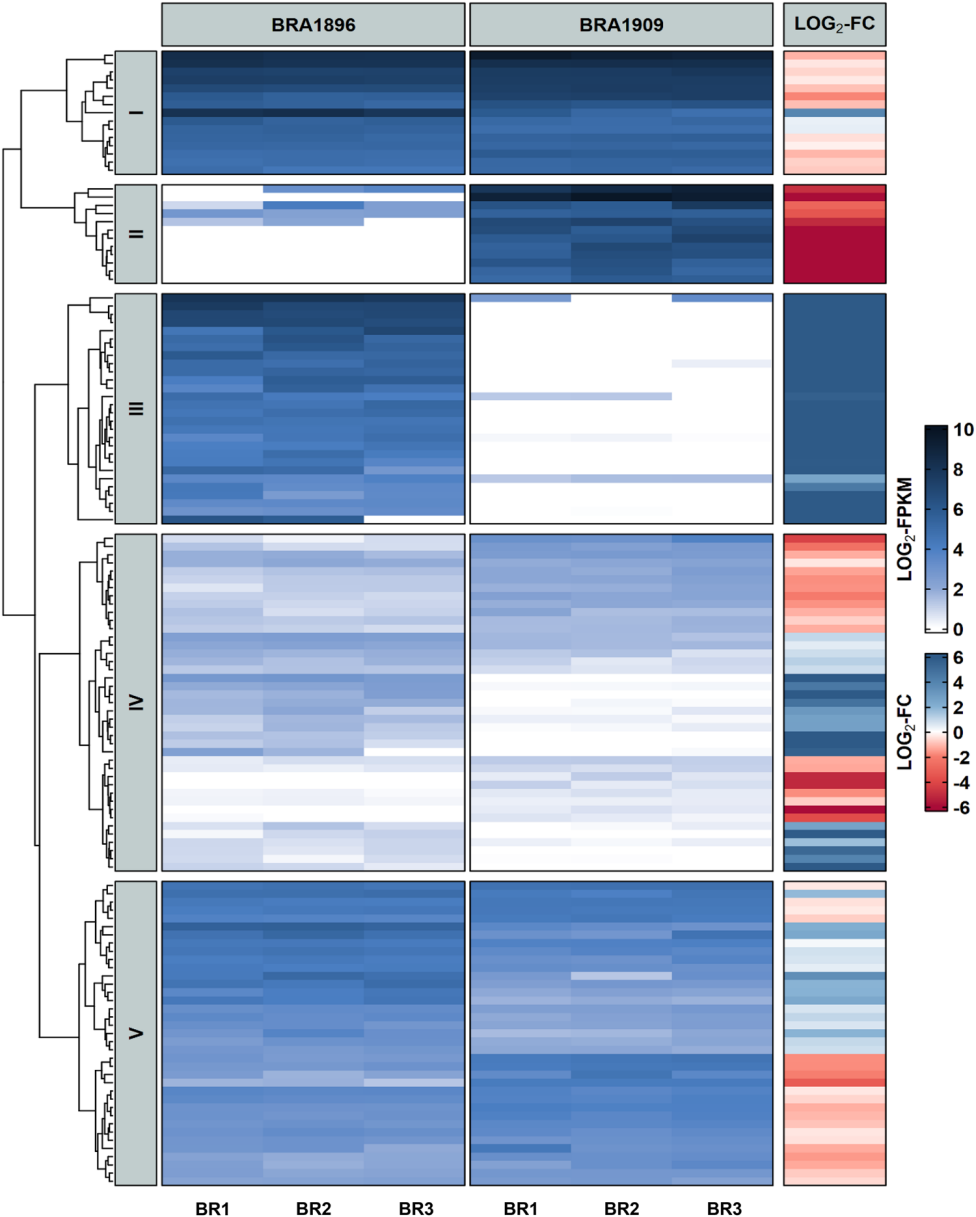
Heatmap of 133 differentially expressed genes (DEGs) related to trichome formation between the hairy *B. villosa* (BRA1896) and the glabrous *B. oleracea* (BRA1909). The genes were automatically subdivided into five clusters (Cluster I–V) based on the log_2_-transformed fragments per million per kilobases (FPKM) expression profiles. The darker the blue the stronger the gene expression. The adjacent heatmap illustrates the log_2_-transformed fold change of the FPKM values between both *Brassica* species. Here, blue means a stronger induction in the hairy *B. villosa* and red means a stronger induction in the glabrous *B. oleracea*. *BR* Biological replication

We screened the RNAseq data to identify candidate genes related to trichome development. Totally, 221 genes were identified, which are related to the GO term “trichome differentiation” (GO:0010026). Of these 133 were identified as differentially expressed genes (DEGs) between the hairy *B. villosa* and the glabrous *B. oleracea* (Table 3). The 221 genes are further divided into GO child terms such as “trichome morphogenesis” (GO:0010090) or “trichome branching” (GO:0010091). By comparison, we found that the 133 DEGs are functionally clustered (Fig. 5). Cluster III, with 28 trichome-associated genes, was exclusively expressed in *B. villosa,* of which 23 genes were out of our de novo assembly and not detectable in the *B. oleracea* TO1000 reference genome, suggesting that they are specific to *B. villosa*. Several of the genes showed homology to trichome-related genes in *A. thaliana* such as *AthXIK*, *AthMYB82*, *AthETD1*, or *AthHDG2* (Table 4).

**Table 3:** Number of trichome-related genes and differentially expressed genes (DEGs) detected in the wild *Brassica* species by RNAseq

Category	Genes	DEGs	Term	Ontology
GO:0010026	221	133	trichome differentiation	BP
GO:0010091	94	57	trichome branching	BP
GO:0048629	2	2	trichome patterning	BP
GO:0010090	178	106	trichome morphogenesis	BP
GO:1,900,032	1	1	regulation of trichome patterning	BP
GO:1,900,033	1	1	negative regulation of trichome patterning	BP
GO:2,000,039	3	2	regulation of trichome morphogenesis	BP
GO:1,905,499	8	3	trichome papilla formation	BP

*DEGs* Differentially expressed genes between *B. villosa* (hairy) and *B. oleracea* (glabrous)

**Table 4:** Expression profiles of trichome-related genes specifically expressed in the hairy *B. villosa* (BRA1896)

Gene	Gene_ID	Chr	Start	End	Ath-ID	Fragments per million per kilobase [FPKM]					
						BRA1896-BR1	BRA1896-BR2	BRA1896-BR3	BRA1909-BR1	BRA1909-BR2	BRA1909-BR3
*TTG1*	TRINITY_DN6108_c0_g1_i6	–	–	–	AT5G24520	256.23	258.74	214.29	5.53	0.00	9.37
*FAS2*	TRINITY_DN15157_c0_g1_i1	–	–	–	AT5G64630	203.66	121.81	124.45	0.00	0.00	0.00
*ATB’ GAMMA*	TRINITY_DN721_c0_g1_i1	–	–	–	AT4G15415	124.42	105.63	120.16	0.00	0.00	0.00
*XIK*	TRINITY_DN5116_c0_g8_i1	–	–	–	AT5G20490	120.74	117.68	99.37	0.00	0.00	0.00
*MYB82*	TRINITY_DN28445_c0_g2_i1	–	–	–	AT5G52600	21.35	66.35	131.27	0.00	0.00	0.00
*MSI1*	TRINITY_DN8046_c0_g1_i2	–	–	–	AT5G58230	33.40	80.81	35.25	0.00	0.00	0.00
*RPS13A*	TRINITY_DN2619_c0_g1_i20	–	–	–	AT4G00100	28.59	66.03	42.45	0.00	0.00	0.00
*ARP2*	TRINITY_DN163_c0_g1_i8	–	–	–	AT3G27000	60.51	34.33	36.11	0.00	0.00	0.00
*RLCKVI_A3*	Unigene.4761	C2	11,777,453	11,778,433	AT5G65530	31.80	27.21	42.47	0.00	0.00	0.29
-	TRINITY_DN438_c0_g2_i11	–	–	–	AT2G46600	31.94	40.72	36.62	0.00	0.00	0.00
*MYB82*	TRINITY_DN28445_c0_g2_i8	–	–	–	AT5G52600	16.01	54.11	53.53	0.00	0.00	0.00
*ARP2*	TRINITY_DN163_c0_g1_i39	–	–	–	AT3G27000	11.46	46.78	24.08	0.00	0.00	0.00
*RSW9*	TRINITY_DN4583_c1_g2_i1	–	–	–	AT5G42080	30.50	17.82	15.54	1.32	1.38	0.00
*BRICK1*	TRINITY_DN8124_c0_g2_i1	–	–	–	AT2G22640	23.69	21.85	36.24	0.00	0.00	0.00
*EDT1*	TRINITY_DN2846_c0_g1_i75	–	–	–	AT1G73360	20.36	28.13	32.25	0.00	0.00	0.00
*RBR1*	TRINITY_DN4767_c0_g1_i8	–	–	–	AT3G12280	25.54	22.20	21.88	0.00	0.00	0.00
*WAVE4*	TRINITY_DN25940_c0_g2_i1	–	–	–	AT2G38440	19.86	20.27	24.52	0.00	0.00	0.00
*MYB82*	Unigene.4850	C2	13,159,285	13,160,362	AT5G52600	11.13	20.68	28.03	0.13	0.21	0.04
*STI*	TRINITY_DN16838_c0_g1_i12	–	–	–	AT2G02480	14.89	16.43	21.85	0.00	0.00	0.00
*GLH1*	TRINITY_DN328_c0_g1_i3	–	–	–	AT1G25540	16.28	29.60	17.03	0.00	0.00	0.00
*HDG2*	TRINITY_DN6148_c0_g1_i2	–	–	–	AT1G05230	16.88	22.16	11.87	0.00	0.00	0.00
*SKD1*	TRINITY_DN6467_c0_g1_i6	–	–	–	AT2G27600	35.17	31.48	5.92	0.00	0.00	0.00
*EDT1*	Unigene.19989	C5	37,708,424	37,710,683	AT1G73360	10.61	10.14	13.80	1.33	1.77	1.78
*WVD2*	TRINITY_DN4022_c0_g3_i5	–	–	–	AT5G28646	19.70	9.25	10.22	0.00	0.00	0.00
*ARP2*	TRINITY_DN163_c0_g1_i14	–	–	–	AT3G27000	18.51	5.03	9.91	0.00	0.00	0.00
*HDG12*	Unigene.2953	C1	38,274,278	38,275,721	AT1G17920	9.80	10.25	10.37	0.00	0.00	0.00
*MYB23*	Unigene.15519	C4	37,455,458	37,458,114	AT5G40330	7.02	7.75	9.34	0.00	0.04	0.00
*MYB82*	TRINITY_DN28445_c0_g2_i9	–	–	–	AT5G52600	72.95	54.90	0.00	0.00	0.00	0.00

^*^Genes with “TRINITY” prefixes were not mappable to the reference genome of *B. oleracea* and assembled de novo

*Chr* Chromosome, *Ath-ID* Ortholog in *A. thaliana*, *BR* Biological replication

According to the reference annotation of the *B. oleracea* TO1000 genome and our RNAseq data, 242 genes are predicted within the peak region of tQTL1 (14.27–16.59 Mbp) and 103 genes within the peak region of tQTL2 (44.31–44.79 Mbp). While the peak region of tQTL3 (2.11–2.82 Mbp) harbors 108 genes, the peak region of tQTL4 (17.86–17.98 Mbp) has 12 genes only (Supplementary Data 3). To narrow down the candidate genes, we first focused on the orthologous transcription factors known to be involved in trichome development in *A. thaliana*. They are the positive regulators *GL1*, *GL2*, *GL3*, *EGL3*, and *TTG1*, and the negative regulator *TRY* (Balkunde et al. 2010). From two orthologs of *GL2*, two of *GL3*, two of *EGL3*, and four of *TTG1*, as well as five orthologs of *TRY* identified in the *B. oleracea* reference genome, only an orthologous gene of *TRY* (*TRYc*, Bo1g051040), referred as to *BoTRYc,* was localized within the tQTL1 on chromosome C01, which is positioned within the peak of tQTL1 at 14.4 Mbp (Fig. 4, Supplementary Table 4). While higher expression levels were observed for orthologous of *GL2* (*GL2a*), *GL3* (*GL3a* and *GL3b*), and *TRY* (*TRYe*) on chromosome C09 in *B. villosa* compared to *B. oleracea,* transcripts of *BoTRYc* were not detectable in our RNAseq data (FPKM = 0). The orthologous *TTG1b* was specifically expressed in *B. oleracea,* while *TTG1c* and *TTG1d* were both highly expressed in *B. villosa* (Supplementary Table 4).

**Fig. 6:**
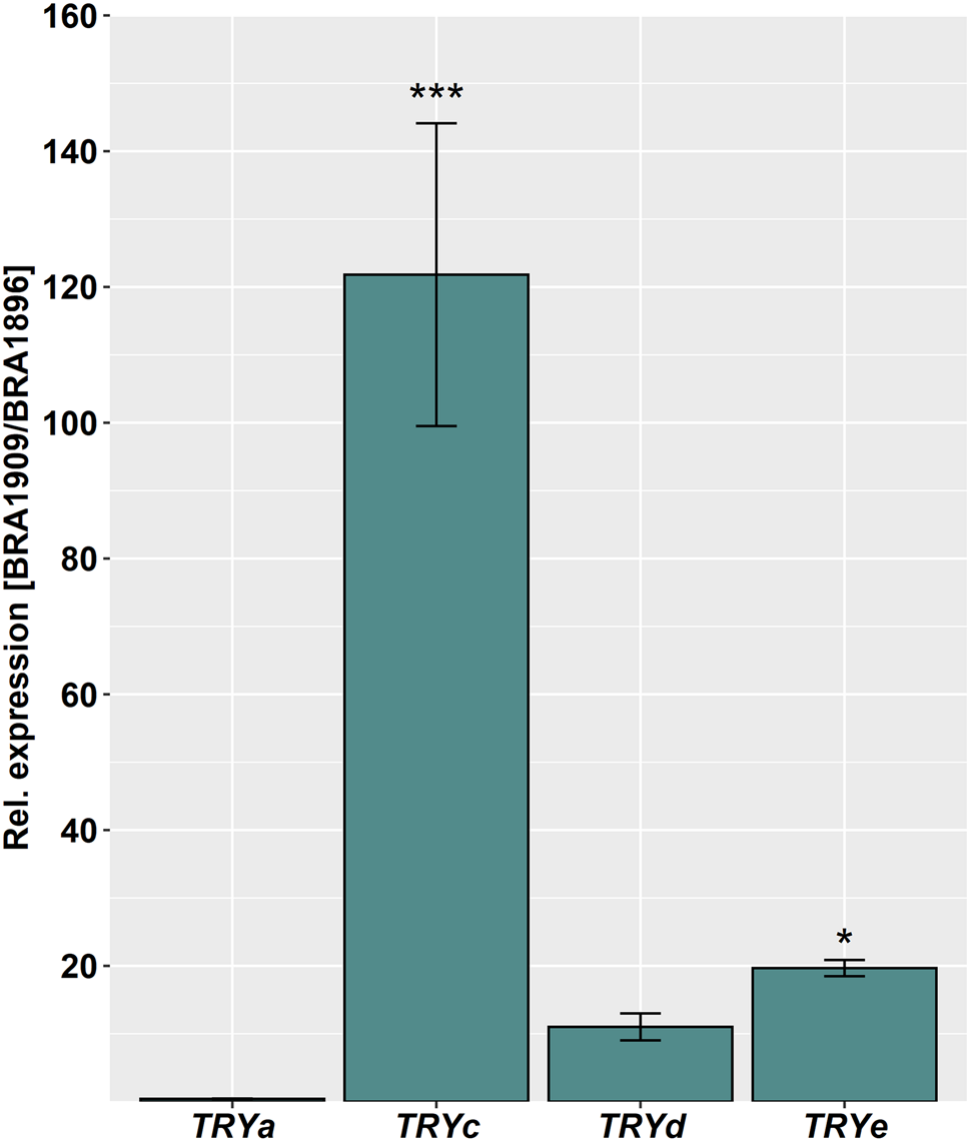
Relative gene expression of *BoTRY* orthologs between the glabrous *B. oleracea* (BRA1909) in comparison to the hairy *B. villosa* (BRA1896) in leaf tissue by real-time quantitative PCR (rt-qPCR). *BoTRY* gene expression was normalized to *BoACT2* and gene expression in *B. oleracea* (BRA1909) was set in relation to *B. villosa* (BRA1896). Error bars represent standard deviation of three biological replications. Asterisks indicate a significantly higher expression in *B. oleracea* compared to *B. villosa* calculated by a linear model using generalized least squares and multiple contrast tests. *P adj. < 0.05, ***P adj. < 0.001

Considering the possibility of tissue-specific expression of these genes, we determined the expression levels of all *TRY* orthologs (*BoTRYa*, b, c, d, e) in leaves of *B. villosa* compared to those of *B. oleracea* by RT-qPCR. As shown in Fig. 6, the *TRY* orthologs, *BoTRYc, BoTRYd, and BoTRYe* are highly expressed in the leaves of *B. oleracea* compared to *B. villosa* with 121.8-fold, 11-fold and 19.7-fold changes, respectively, whereas only *BoTRYa,* to contrast, showed a 0.4-fold lower expression level in *B. oleracea* than in *B. villosa* (Fig. 6). *BoTRYb* could not be detectable in leaves of both *Brassica* species.

## Discussion

### Trichome formation in *B. villosa* is controlled by multiple genetic loci

Phenotypic assessment of hairiness in the F_2_ population of *B. villosa* x *B. oleracea* showed a continuous variation from hairless to hairy individuals, indicating a quantitative trait controlled by multiple genes. In *B. incana*, hairiness segregated 3:1 (glabrous/hairy) in a F_2_ population with dominance of the glabrous allele (Mei et al. 2017). Trichome mapping studies in *Brassica rapa* showed Mendelian inheritance patterns with the trichome trait being dominant (Ye et al. 2016; Zhang et al. 2021; Li et al. 2022). The different inheritance of hairiness between *B. villosa* and diverse *Brassica* species is most likely caused by different phenotyping assessments. Mei et al. (2017) examined only leaf trichomes of an F_2_ population and classified individuals into two groups: hairy and non-hairy. In this study, we used a finer phenotyping scale to distinguish between hairy leaves and petioles and their trichome density and detected four QTLs for hairiness in *B. villosa*. Our data suggest that the leaf trichome is likely mainly controlled by a single dominant gene in *B. oleracea* as reported in *B. rapa* (Ye et al. 2016; Zhang et al. 2021; Li et al. 2022). In *B. juncea*, the leaf trichome trait was dominant but showed a more complex inheritance (Meng et al. 2023), in which 196 F_2_ individuals out of 1304 F_2_ individuals were glabrous and hairy individuals followed a continuous frequency distribution of trichome phenotypes. Although major loci for leaf trichomes in *B. rapa* have been reported by various groups (Ye et al. 2016; Zhang et al. 2021; Li et al. 2022), genetic studies on trichomes in the cytodeme of *B. oleracea* are still rare, as most major cultivars of *B. oleracea* are glabrous (Mei et al. 2017). By using a finer phenotyping scale, we provide a deep insight into the genetic architecture of trichomes in *B. villosa*.

### *BoTRY* is a major candidate for trichome development variation in *Brassica* species

The finding that the major QTL on chromosome C01 (t1QTL) in this study overlaps with the locus for leaf trichomes in the wild *B. incana* reported by Mei et al. (2017) supports that genetic factors controlling trichome development may be conserved in relatives of the *B. oleracea* cytodeme. Like Mei et al. (2017), we identified *BoTRY*, a transcription factor negatively regulating trichome development, as a candidate gene. Unexpectedly, transcriptomic analysis by RNAseq in petiole tissue did not find the transcripts of *BoTRY,* neither in the hairy *B. villosa* nor in the glabrous *B. oleracea*. Mei et al. (2017) have reported a low-level expression of *BoTRY* in hairy plants but a significantly higher expression of *BoTRY* in glabrous plants. By contrast, a high expression of *BoTRY* was reported in haired *B. villosa* subsp. *drepanensis* by Nayidu et al. (2014a). The discrepancy in the expression of *BoTRY* in different studies may be due to structural variation within this locus between the reference genome and the analyzed *Brassica* species, which hinders a correct transcript assembly and its quantification. Structural variation was identified as a key factor in *BrGL1* (Li et al. 2011) and *BrTTG1* (Zhang et al. 2009) for hairiness in *B. rapa*. Alternatively, it is also reasonable to speculate that *BoTRY* is a leaf-specific gene (Mei et al. 2017) and not or is less expressed in petiole tissue. Following this, we determined the gene expression levels of *BoTRY* orthologues in leaf tissue with RT-qPCR and demonstrated that three *BoTRY* orthologues were highly expressed in *B. oleracea* compared to *B. villosa*. Strikingly, *BoTRYc* (Bo1g051040) located in the tQTL1 on chromosome C01 showed a 121.8-fold stronger expression in *B. oleracea* than in *B. villosa.* This is in line with the findings made by Mei et al. (2017), supporting that *BoTRYc* might be a master-switch regulator of leaf trichome development in the cytodeme of *B. oleracea*. Nevertheless, the contradictory *BoTRY* expression patterns across different *Brassica* species and tissue reflect the genetic complexity of trichome development in the genus *Brassica,* despite the presence of common genetic factors. Natural variation in the regulators of trichome development, as observed in *BviTRY* (Nayidu et al. 2014a), could be a driving force behind the increased trichome density and be, therefore, responsible for the complex expression patterns of *TRY* orthologues across the wild *Brassica* species (Ishida et al. 2008).

Although 223 genes associated with trichome development were identified, of which 133 were differentially expressed between the hairy and the glabrous *Brassica* species by transcriptomic analysis, the identification of genes for trichome development in this study is somewhat hampered, as many genes within the QTLs have not yet been annotated. Also the RNAseq data generated with petioles from our previous study for Sclerotinia-resistance (Bergmann et al. 2023) may also be subject to restrictions for this study, as we only obtained genes expressed in petioles at a single time point, but not those differentially expressed in different developmental stages or tissues, for example, in leaves. Nevertheless, we obtained several clusters of candidate genes for trichome development in this study, offering opportunities to decipher the mechanisms of trichome development in *Brassica* species. Further analyses, such as genome re-sequencing, are needed to characterize e.g. structural variants of the *BoTRY* locus and their functional relevance. To our knowledge, the QTLs for trichomes on chromosomes C03, C04 and C09 have not yet been reported in *B. oleracea*, so the data from this study provide new genetic sources for breeders to develop molecular markers.

### Association between hairy genotypes and Sclerotinia-resistance

Noticeably, the LOD peak of the major locus t1QTL pointed directly to l2QTLb, a major QTL for Sclerotinia-resistance (Bergmann et al. 2023). This provoked us to investigate a functional association of hairy genotypes with Sclerotinia-resistance. Statistical analyses revealed a correlation between lower lesion values and trichomes across the experiments and assays, except for the 3rd petiole assay, this correlation was, however, weak as the explainable variance was low across all assays. It is likely that the co-localization of both major QTLs is merely a physical overlap rather than sharing key genetic factors. The stronger Spearman’s correlations (*P* values < 0.02) observed in the leaf tests were attributed to the biased infection results caused by the effect of trichomes on the inoculation process. For the detached-leaf assay, the Sclerotinia-plugs were placed directly on the leaves with or without trichomes. Since trichomes may physically prevent the contact between the Sclerotinia-plug and the leaf surface, fungal infection could be somehow reduced, leading to a smaller lesion size. In consistency, lower Spearman’s correlations (*P* values < 0.1) could be observed in the detached petiole assays, where the plug with the mycelia was attached directly to the open petiole without trichomes. Nevertheless, it is reasonable to believe that the trichome layer of *B. villosa* can functionally contribute to defence against *Sclerotinia* via conferring a chemical barrier. An accumulation of metals and other metabolic compounds in trichomes has been reported in the closely related *B. villosa* subsp. *drepanensis* (Nayidu et al. 2014b). Thus, further studies are needed to shed more light on the mechanisms.

## Conclusions

In this study, we identified four QTLs for trichome development in *B. villosa*, a hairy *Brassica* species from the C cytodeme of *B. oleracea*. Of these, one major QTL was identified on chromosome C01 in the *B. oleracea* genome overlapping with a QTL for leaf-trichomes detected in *B. incana* (Mei et al. 2017)*,* where *BoTRY,* a candidate gene for trichome development, is located, suggesting a mechanism conserved in *Brassica*. Furthermore, the genetic, genomic, and transcriptomic data from this study provide valuable resources to get deep insights into the genetic architecture of trichome development in *B. villosa* and to decipher underlying molecular mechanisms as well. Molecular markers can be developed to facilitate the introgression and selection of this important trait in oilseed rape breeding, making *B. villosa* even more unique and valuable.

## Supplementary Information

Below is the link to the electronic supplementary material.

Supplementary file1 (DOCX 38 KB)

Supplementary file2 (DOCX 204 KB)

Supplementary file3 (PDF 53 KB)

Supplementary file4 (XLSX 11074 KB)

## Data Availability

The main data generated or analyzed in this study is included in the manuscript and in the supplementary information files, as well as in the manuscript from Bergmann et al. (2023). The raw RNA sequencing data is available at the NCBI Sequence Read Archive (PRJNA706136). Additional data is available on reasonable request.
