# Supplementary material for: Identification of Quantitative Trait Loci (QTLs) and candidate genes for trichome development in Brassica villosa using genetic, genomic, and transcriptomic approaches
Source: Mol Genet Genomics. 2025 Jan 7;300(1):13. doi: 10.1007/s00438-024-02223-5 (PMC11703928; doi:10.1007/s00438-024-02223-5)
Supplement: Supplementary file 1 — Supplementary file1 (DOCX 38 KB) [file 438_2024_2223_MOESM1_ESM.docx]

| **Supplementary Table 1** Summary of the genetic map | | | | |
| --- | --- | --- | --- | --- |
| **Linkage group** | **Marker** | **Length [cM]** | **Ø-Spacing [cM]** | **Max. gap [cM]** |
| C01 | 78 | 66.42 | 0.86 | 8.04 |
| C02 | 100 | 93.71 | 0.95 | 17.34 |
| C03 | 209 | 125.19 | 0.60 | 5.36 |
| C04a | 35 | 28.63 | 0.84 | 3.18 |
| C04b | 53 | 49.04 | 0.94 | 8.10 |
| C05 | 107 | 92.53 | 0.87 | 5.68 |
| C06 | 103 | 77.67 | 0.76 | 5.35 |
| C07 | 113 | 84.60 | 0.76 | 5.34 |
| C08 | 104 | 83.27 | 0.81 | 7.67 |
| C09 | 68 | 110.74 | 1.65 | 18.47 |
| **overall** | **970** | **811.8** | **0.8** | **18.5** |
| *cM* centiMorgan | | | | |

| **Supplementary Table 2** Stepwise Williams' trend tests between trichome groups and the lesion values | | | | | | | |
| --- | --- | --- | --- | --- | --- | --- | --- |
| **Experiment** | **Assay** | **Group comparison*** | **Estimate** | **Std. error** | **t value** | ***P* value** | **signif. Code** |
| 1^st^ | Leaf | C 1 == 0 | -57.200 | 28.770 | -1.988 | **0.097** | **.** |
|  |  | C 2 == 0 | -56.190 | 26.290 | -2.137 | **0.071** | **.** |
|  |  | C 3 == 0 | -45.770 | 23.630 | -1.937 | 0.108 |  |
|  |  | C 4 == 0 | -38.010 | 21.820 | -1.742 | 0.159 |  |
| 2^nd^ | Leaf | C 1 == 0 | -199.010 | 41.510 | -4.794 | **< 0.001** | ******* |
|  |  | C 2 == 0 | -165.110 | 38.340 | -4.306 | **< 0.001** | ******* |
|  |  | C 3 == 0 | -120.800 | 34.130 | -3.54 | **0.00** | ****** |
|  |  | C 4 == 0 | -99.760 | 31.220 | -3.195 | **0.00** | ****** |
| 3^rd^ | Leaf | C 1 == 0 | -34.403 | 30.151 | -1.141 | 0.427 |  |
|  |  | C 2 == 0 | -13.636 | 27.550 | -0.495 | 0.872 |  |
|  |  | C 3 == 0 | -5.590 | 24.762 | -0.226 | 0.988 |  |
|  |  | C 4 == 0 | -2.341 | 22.910 | -0.102 | 0.999 |  |
| 1^st^ | Petiole | C 1 == 0 | -2.217 | 1.118 | -1.983 | **0.098** | **.** |
|  |  | C 2 == 0 | -2.092 | 1.022 | -2.048 | **0.086** | **.** |
|  |  | C 3 == 0 | -0.910 | 0.918 | -0.991 | 0.523 |  |
|  |  | C 4 == 0 | -0.454 | 0.848 | -0.536 | 0.846 |  |
| 2^nd^ | Petiole | C 1 == 0 | -3.481 | 1.428 | -2.437 | **0.035** | ***** |
|  |  | C 2 == 0 | -2.248 | 1.308 | -1.718 | 0.168 |  |
|  |  | C 3 == 0 | -1.054 | 1.166 | -0.904 | 0.588 |  |
|  |  | C 4 == 0 | -1.086 | 1.067 | -1.017 | 0.511 |  |
| 3^rd^ | Petiole | C 1 == 0 | 0.291 | 1.034 | 0.281 | 0.975 |  |
|  |  | C 2 == 0 | 1.461 | 0.945 | 1.546 | 0.226 |  |
|  |  | C 3 == 0 | 1.567 | 0.855 | 1.832 | 0.133 |  |
|  |  | C 4 == 0 | 1.604 | 0.789 | 2.032 | **0.087** | **.** |
| *Contrasts: | C 1 == 0 | Trichome groups: | "4" vs "0" | | | | |
|  | C 2 == 0 |  | "4"+"3" vs "0" | | | | |
|  | C 3 == 0 |  | "4"+"3"+"2" vs "0" | | | | |
|  | C 4 == 0 |  | "4"+"3"+"2"+"1" vs "0" | | | | |

| **Supplementary Table 3** Comparison of the lesion values between the trichome groups to the grand mean | | | | | | | |
| --- | --- | --- | --- | --- | --- | --- | --- |
| **Experiment** | **Assay** | **Mean comparison*** | **Estimate** | **Std. error** | **t value** | ***P* value** | **signif. Code** |
| 1^st^ | Leaf | "0" == 0 | 22.449 | 12.887 | 1.742 | 0.322 |  |
|  |  | "1" == 0 | 2.814 | 23.259 | 0.121 | 1 |  |
|  |  | "2" == 0 | -1.571 | 27.215 | -0.058 | 1 |  |
|  |  | "3" == 0 | -30.733 | 39.052 | -0.787 | 0.906 |  |
|  |  | "4" == 0 | -34.747 | 20.776 | -1.672 | 0.362 |  |
| 2^nd^ | Leaf | "0" == 0 | 58.194 | 18.211 | 3.195 | **0.00816** | ****** |
|  |  | "1" == 0 | 3.405 | 32.209 | 0.106 | 0.99996 |  |
|  |  | "2" == 0 | 23.912 | 39.025 | 0.613 | 0.96028 |  |
|  |  | "3" == 0 | 13.612 | 62.204 | 0.219 | 0.99921 |  |
|  |  | "4" == 0 | -140.813 | 30.298 | -4.648 | **< 0.001** | ******* |
| 3^rd^ | Leaf | "0" == 0 | 1.377 | 13.476 | 0.102 | 1 |  |
|  |  | "1" == 0 | 6.991 | 24.862 | 0.281 | 0.998 |  |
|  |  | "2" == 0 | 12.577 | 28.505 | 0.441 | 0.988 |  |
|  |  | "3" == 0 | 50.043 | 40.913 | 1.223 | 0.662 |  |
|  |  | "4" == 0 | -33.026 | 21.753 | -1.518 | 0.46 |  |
| 1^st^ | Petiole | "0" == 0 | 0.2682 | 0.5007 | 0.536 | 0.9756 |  |
|  |  | "1" == 0 | 0.892 | 0.9037 | 0.987 | 0.8106 |  |
|  |  | "2" == 0 | 1.8258 | 1.0574 | 1.727 | 0.3309 |  |
|  |  | "3" == 0 | -1.4463 | 1.5174 | -0.953 | 0.8291 |  |
|  |  | "4" == 0 | -1.9491 | 0.8072 | -2.415 | **0.0764** | **.** |
| 2^nd^ | Petiole | "0" == 0 | 0.6282 | 0.6177 | 1.017 | 0.7933 |  |
|  |  | "1" == 0 | -0.5262 | 1.116 | -0.471 | 0.9847 |  |
|  |  | "2" == 0 | 1.9614 | 1.3512 | 1.452 | 0.5048 |  |
|  |  | "3" == 0 | 2.3266 | 2.0347 | 1.143 | 0.7151 |  |
|  |  | "4" == 0 | -2.8528 | 1.0501 | -2.717 | **0.0348** | ***** |
| 3^rd^ | Petiole | "0" == 0 | -0.9355 | 0.4603 | -2.032 | 0.1847 |  |
|  |  | "1" == 0 | 0.755 | 0.8518 | 0.886 | 0.8631 |  |
|  |  | "2" == 0 | 0.875 | 1.0293 | 0.85 | 0.8799 |  |
|  |  | "3" == 0 | 4.0357 | 1.4027 | 2.877 | **0.0218** | ***** |
|  |  | "4" == 0 | -0.6448 | 0.745 | -0.866 | 0.8729 |  |
| *0 = grand mean of all trichome groups | | | | | | | |

**Supplementary Table 4** Expression profiles of trichome-related candidate genes in petiole tissue of B. villosa (BRA1896) and B. oleracea (BRA1909)

| **Gene** | **Gene_ID** | **Chr** | **Start** | **End** | **Ath-ID** | **Fragments per million per kilobase [FPKM]** | | | | | |
| --- | --- | --- | --- | --- | --- | --- | --- | --- | --- | --- | --- |
|  |  |  |  |  |  | **BRA1896-1** | **BRA1896-2** | **BRA1896-3** | **BRA1909-1** | **BRA1909-2** | **BRA1909-3** |
| *GL2a* | Unigene.22583 | C6 | 24183324 | 24187131 | AT1G79840 | 5.04 | 4.89 | 4.88 | 0.04 | 0.00 | 0.00 |
| *GL2b* | TRINITY_DN7884_c0_g1_i1 | - | - | - | AT1G79840 | 0.00 | 0.00 | 0.00 | 0.00 | 0.00 | 0.00 |
| *GL3a* | Bo4g141980 | C4 | 38327546 | 38328319 | AT5G41315 | 0.89 | 0.00 | 0.53 | 0.00 | 0.11 | 0.00 |
| *GL3b* | Bo4g141990 | C4 | 38330554 | 38332853 | AT5G41315 | 0.37 | 0.45 | 0.49 | 0.12 | 0.21 | 0.15 |
| *EGL3a* | Bo9g029230 | C9 | 10214709 | 10217809 | AT1G63650 | 0.18 | 0.19 | 0.00 | 0.21 | 0.35 | 0.11 |
| *EGL3b* | Unigene.32857 | C9 | 11928773 | 11933805 | AT1G63650 | 0.18 | 0.66 | 1.01 | 3.50 | 1.75 | 1.81 |
| *TTG1a* | Unigene.26631 | C7 | 37466122 | 37467693 | AT5G24520 | 1.61 | 41.92 | 34.97 | 42.39 | 46.17 | 49.61 |
| *TTG1b* | TRINITY_DN6108_c0_g1_i3 | - | - | - | AT5G24520 | 0.00 | 0.00 | 0.00 | 554.52 | 910.27 | 682.44 |
| *TTG1c* | TRINITY_DN6108_c0_g1_i4 | - | - | - | AT5G24520 | 287.68 | 338.63 | 240.34 | 58.98 | 35.44 | 26.71 |
| *TTG1d* | TRINITY_DN6108_c0_g1_i6 | - | - | - | AT5G24520 | 256.23 | 258.74 | 214.29 | 5.53 | 0.00 | 9.37 |
| *TRYa* | Bo3g022870 | C3 | 8058767 | 8059844 | AT5G53200 | 0.00 | 0.00 | 0.14 | 0.00 | 0.00 | 0.11 |
| *TRYb* | Unigene.28874 | C8 | 17644376 | 17648699 | AT5G53200 | 0.13 | 0.00 | 0.00 | 0.00 | 0.20 | 0.00 |
| *TRYc* | Bo1g051040 | C1 | 14433200 | 14433768 | AT5G53200 | 0.00 | 0.00 | 0.00 | 0.00 | 0.00 | 0.00 |
| *TRYd* | Bo2g046050 | C2 | 12552624 | 12553806 | AT5G53200 | 0.25 | 0.08 | 0.00 | 0.21 | 0.00 | 0.49 |
| *TRYe* | Unigene.34109 | C9 | 35425590 | 35426838 | AT5G53200 | 0.98 | 2.18 | 1.27 | 0.05 | 0.12 | 0.34 |

*Chr* Chromosome, *Ath-ID* Ortholog in *A. thaliana*

**Supplementary Table 5** Primer used in this study

| **Gene** | **Gene ID** | **Primer** | **Oligo** | **Orientation** | **Length [bp]** | **Tmp [°C]** | **GC [%]** | **Amplicon [bp]** |
| --- | --- | --- | --- | --- | --- | --- | --- | --- |
| *TRYd* | Bo2g046050 | Bo2g046050_fw | GAAGACTCTGAAGAAGTGAGCAGC | fw | 24 | 57.5 | 0.5 | 223 |
|  |  | Bo2g046050_rev | GAGAGGAAGAGTGGTGAAGTTGCA | rev | 24 | 58.8 | 0.5 |  |
| *TRYa* | Bo3g022870 | Bo3g022870_fw | CCATGACTCTGAAGAAGTGAGCAGT | fw | 25 | 58.7 | 0.48 | 191 |
|  |  | Bo3g022870_rev | CCGCAAAGCCATCACTATTTCTCATT | rev | 26 | 58.2 | 0.42 |  |
| *TRYe* | Unigene.34109 | Unigene.34109_fw | TAAGCAACACAAAGCCACTCTCCAT | fw | 25 | 58.8 | 0.44 | 213 |
|  |  | Unigene.34109_rev | CATTCAATACTGCTCACTTTTAGCACGG | rev | 28 | 58.8 | 0.43 |  |
| *TRYc* | Bo1g051040 | Bo1g051040_fw | CGACGTCAGCGTCACAACTC | fw | 22 | 58.6 | 0.5 | 113 |
|  |  | Bo1g051040_rev | CTATCGCCTACGAGTCTGTACATTCT | rev | 26 | 57.8 | 0.46 |  |
| *TRYb* | Unigene.28874 | Unigene.28874_fw | GAATGCAGAAGTGTGTAGTGTGGAATG | fw | 27 | 58.6 | 0.44 | 168 |
|  |  | Unigene.28874_rev | GGAAGATGTGTGTGTTCTTTCTCACG | rev | 26 | 58.7 | 0.46 |  |
