# Supplementary material for: Identification of Quantitative Trait Loci (QTLs) and candidate genes for trichome development in Brassica villosa using genetic, genomic, and transcriptomic approaches
Source: Mol Genet Genomics. 2025 Jan 7;300(1):13. doi: 10.1007/s00438-024-02223-5 (PMC11703928; doi:10.1007/s00438-024-02223-5)
Supplement: Supplementary file 2 — Supplementary file2 (DOCX 204 KB) [file 438_2024_2223_MOESM2_ESM.docx]

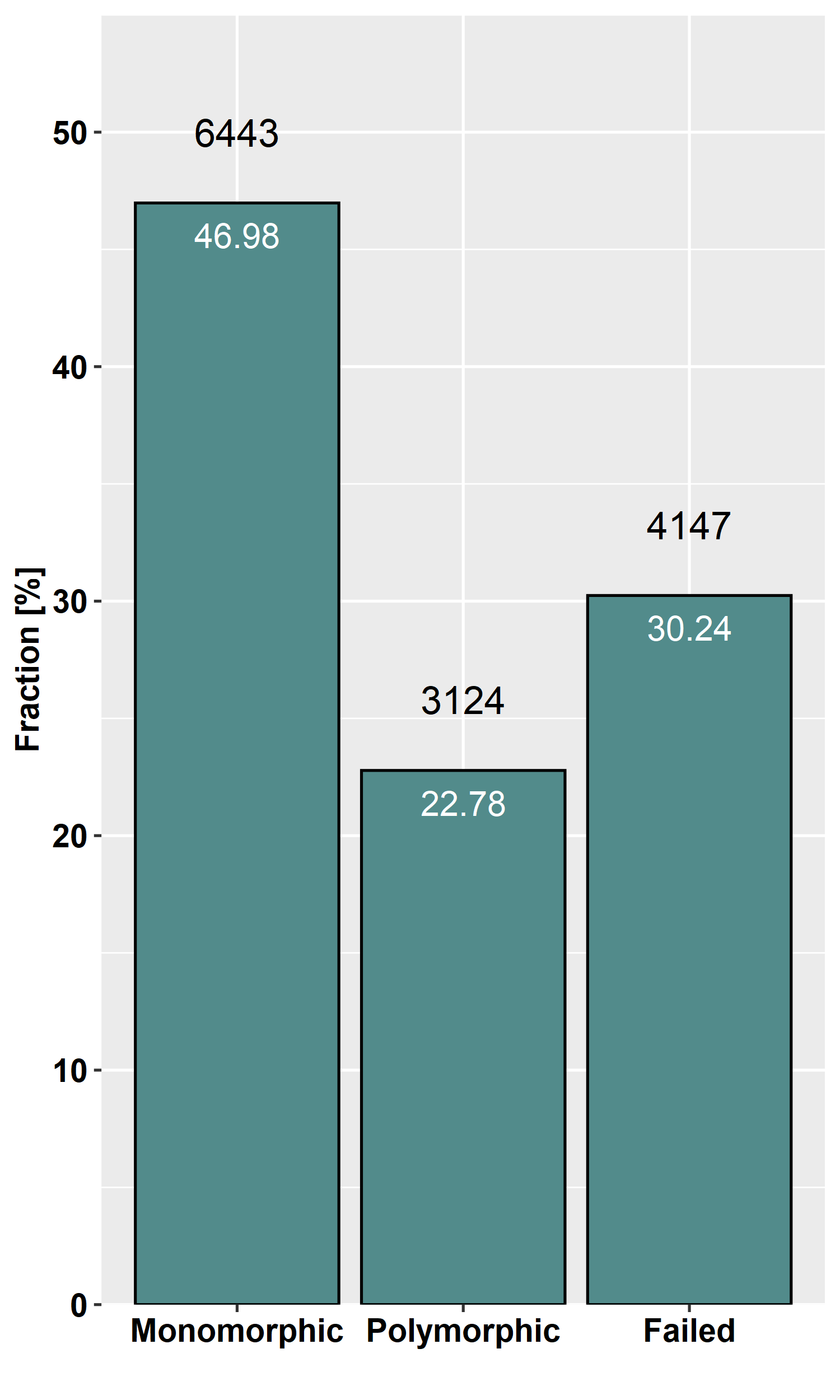
**Supplementary Fig. 1** Fraction of monomorphic, polymorphic, and failed single nucleotide polymorphism (SNP) markers from the 15k Illumina Brassica Infinium Array for the F_2_ population.


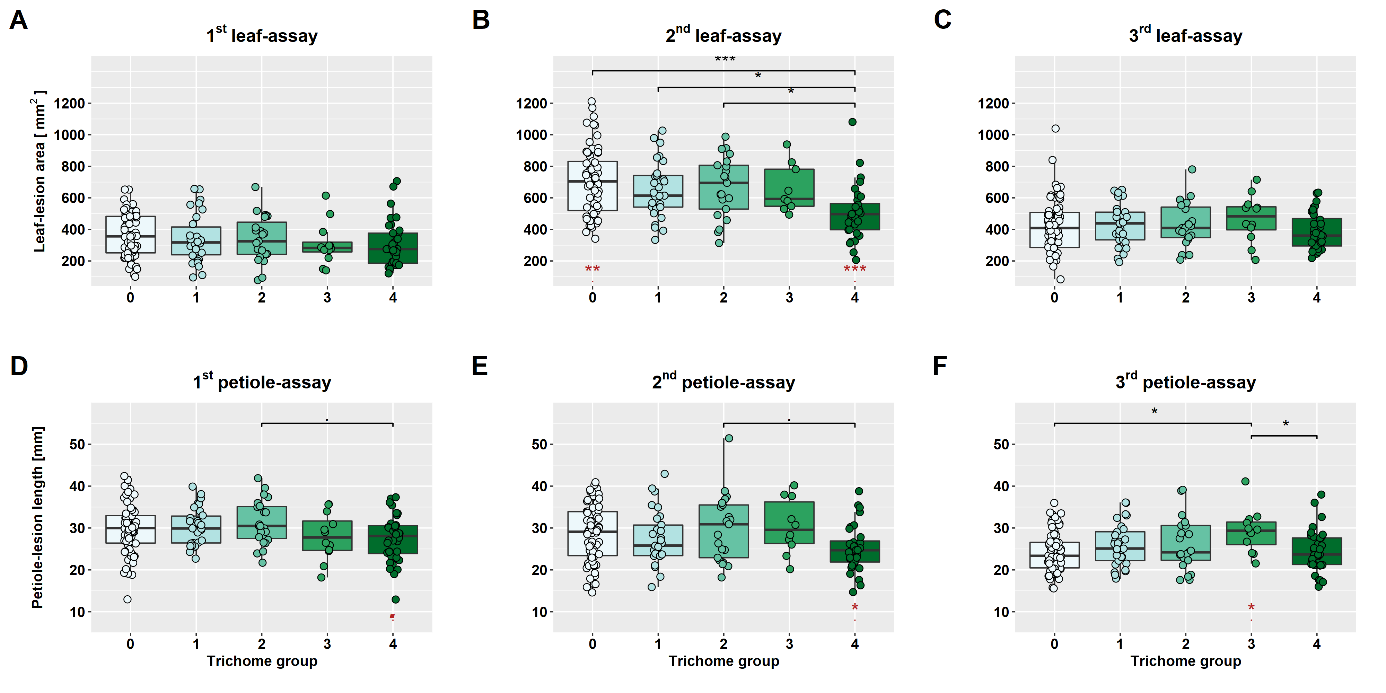


**Supplementary Fig. 2** Leaf- and petiole-lesion comparisons of the trichome groups after infection with S. sclerotiorum. Lesion values were adopted from Bergmann et al. 2023. For each F_2_ plant and assay, two leaves and two petioles were infected with Sclerotinia, and the lesion sizes were averaged. **A – C** Lesion sizes of the leaf-assay with three replications. **D – F** Lesion sizes of the petiole-assay with three replications. Trichome groups were compared in each assay and replication by a type III one-way ANOVA followed by Tukey’s all-pair comparison and a comparison to the grand mean of all trichome groups. Significant group differences are highlighted by brackets at the top of the plots and significant deviations from the grand mean are highlighted by red asterisks at the bottom of the plots. Signif. codes: < 0.001 ***, < 0.01 **, < 0.05 *, < 0.1**^·^**
