# Supplementary figures and images for: Identification of Quantitative Trait Loci (QTLs) and candidate genes for trichome development in Brassica villosa using genetic, genomic, and transcriptomic approaches
Source: Mol Genet Genomics. 2025 Jan 7;300(1):13. doi: 10.1007/s00438-024-02223-5 (PMC11703928; doi:10.1007/s00438-024-02223-5)

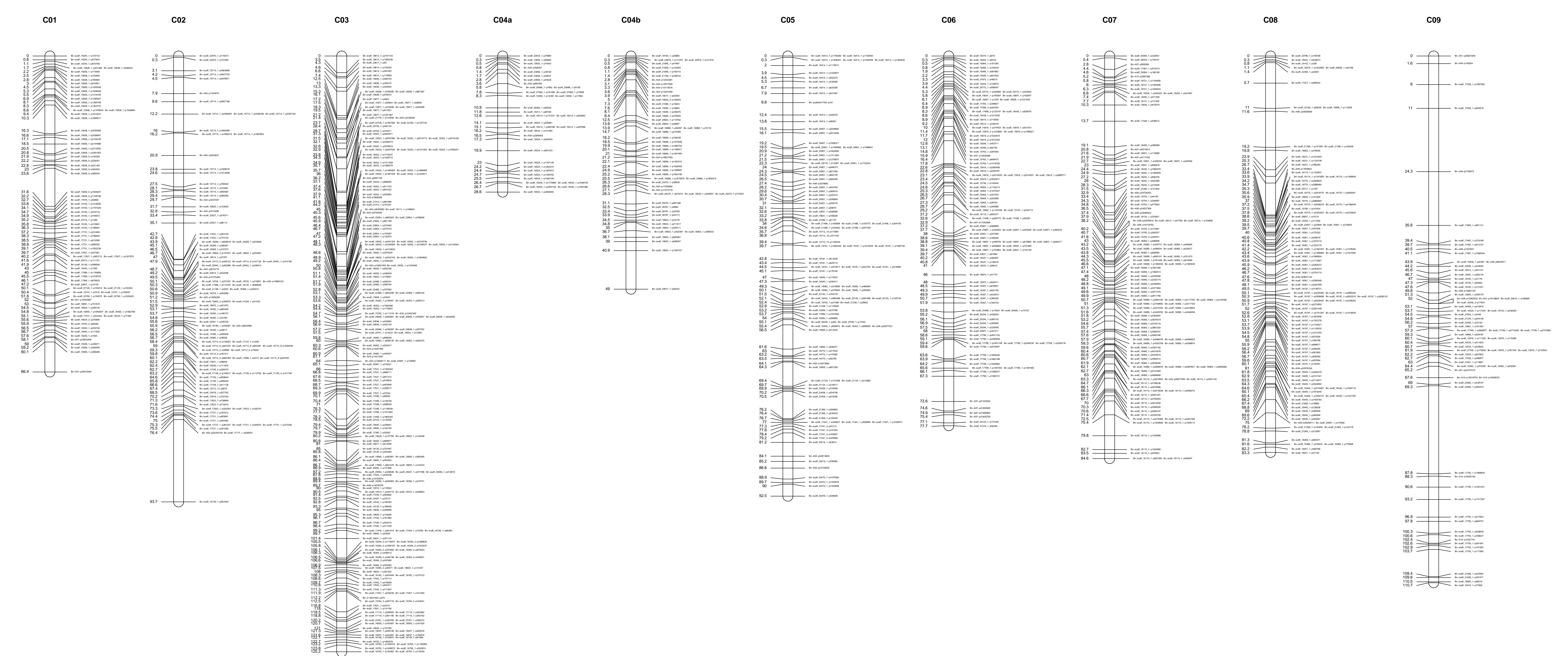

Supplement: Supplementary file 3 — Supplementary file3 (PDF 53 KB) [file 438_2024_2223_MOESM3_ESM.pdf]
